# Remote electrical neuromodulation (REN) wearable device for adolescents with migraine: a real-world study of high-frequency abortive treatment suggests preventive effects

**DOI:** 10.3389/fpain.2023.1247313

**Published:** 2023-11-06

**Authors:** Teshamae S. Monteith, Alit Stark-Inbar, Sharon Shmuely, Dagan Harris, Sandy Garas, Alon Ironi, Paige Kalika, Samantha L. Irwin

**Affiliations:** ^1^ Miller School of Medicine, University of Miami, Miami, FL, United States; ^2^ Clinical Development Department, Theranica, Netanya, Israel; ^3^ Department of Neurology, UCSF Benioff Children’s Hospitals, San Francisco, CA, United States

**Keywords:** adolescents, headache, migraine, prevention, wearable, remote electrical neuromodulation, REN

## Abstract

**Introduction:**

Migraine is a chronic neurological disease manifesting as attacks of disabling head pain and associated symptoms. Remote electrical neuromodulation (REN) is a non-pharmacological, prescribed, wearable device (Nerivio®). This device has been certified by the FDA for the acute and/or preventive treatment of migraine with or without aura in patients 12 years of age or older. The device is affixed to the user’s arm during 45-min treatment sessions and is operated using a smartphone app. This study (NCT05769322) aims to evaluate whether frequent use of REN for the acute treatment of migraine in adolescents resulted in a reduction in monthly migraine treatment days (MMTD), as previously demonstrated in adults through a dedicated prevention clinical trial (NCT04828707).

**Methods:**

The study included real-world prospective data from adolescent patients who used REN on at least 10 days every 28-day month, following the REN migraine prevention guideline of an every-other-day pattern. Additional requirements were at least three REN treatment days in each of the two subsequent months. The number of MMTD was used as a proxy measure for the number of monthly migraine days (MMD). The change in MMTD from the first month, taken as a “baseline,” to each of the following months was used to evaluate the presence and size of potential migraine preventive benefits of REN in adolescents.

**Results:**

A total of 83 adolescents were eligible for analysis. The users were 15.9 ± 1.3 years of age (mean ± SD), and 89% of them were female. The results demonstrated a substantial month-to-month reduction in the mean (±SD) number of REN treatment days from 12.6 (±3.2) MMTD in the first month to 9.0 (±4.8) MMTD in the second month (*p* < 0.001), and a further decrease to 7.4 (±4.2) MMTD in the third month (*p* < 0.001). This indicates an accumulative reduction of 5.2 (±4.8) mean REN MMTD from the first month to the third month of consecutive REN treatment. The users also reported consistent 2-h acute pain responses in at least 50% of their treated attacks, with 61.9% of the users reported experiencing pain relief, 24.5% reported pain freedom, 67.4% indicated relief in functional disability, and 41.3% reported complete freedom from functional disability.

**Conclusion:**

The frequent use of REN among adolescents as an acute treatment for migraine attacks resulted in a decrease in the mean number of monthly treatment days in the subsequent months, suggesting that REN may have potential preventive benefits for migraine in this subpopulation.

## Introduction

Migraine is the second most disabling disease among adults worldwide. It is a chronic neurological disease manifesting as recurrent attacks of moderate-to-severe throbbing and disabling head pain, dysfunction in sensory perception, nausea or vomiting, and emotional-cognitive disturbances (1). Migraine is the predominant form of disabling headache in children and adolescents, with a mean prevalence rate of approximately 10%, and several studies have shown prevalence rates even up to 23% (2–5). Migraine is a significant cause of disability in the pediatric population, particularly in those experiencing frequent migraine attacks, encompassing 0.8%–1.8% of adolescents who meet the criteria for chronic migraine (6). Studies on measuring the quality of life (QoL) showed that the impact of severe migraine on children is similar to that of diabetes, arthritis, and cancer (6). Children diagnosed with migraine self-report greater levels of impairment in both school functioning and emotional functioning compared with children diagnosed with other chronic illnesses, possibly attributed to the unpredictable and disruptive nature of migraine attacks (7).

The significant negative effect of high-frequency migraine on adolescents necessitates the urgent evaluation of novel treatment options aimed at reducing the frequency of attacks and the resulting headache-related disability in this vulnerable population. According to practice guidelines for pediatric migraine prevention established by The American Academy of Neurology (AAN) and American Headache Society (AHS), it is recommended that preventive treatment be taken into consideration when headaches occur with sufficient frequency and severity, or when they lead to migraine-related disability (2). However, there is a paucity of highly effective, evidence-based preventive treatment options for migraine in children and adolescents. In addition, conducting randomized controlled trials in the pediatric population is challenging due to the notable prevalence of placebo responses (6).

Remote electrical neuromodulation (REN) is a non-pharmacological, non-invasive, acute and/or preventive treatment for migraine with or without aura, cleared by the FDA for patients aged 12 and above. It is a prescription-based, self-administered device (Nerivio®) designed to be worn on the upper arm for 45-min treatment sessions and is operated through a smartphone app. It is indicated for use in the home environment at the onset of migraine headache or aura for acute treatment, or follow every-other-day protocol for preventive treatment.

Multiple studies have shown that REN is a safe, tolerable, and efficacious treatment of episodic or chronic migraine (8). Randomized, double-blind, placebo-controlled trials in adults have shown that REN is safe and efficacious for the acute (9) and preventive (10) treatment of migraine, followed by additional clinical studies (11, 12) and real-world evidence research in adults (13, 14). In adolescents, REN was shown to be highly effective, well-tolerated, and safe in a clinical trial evaluating the acute treatment of migraine (15). In addition to the high safety profile, a recent real-world analysis of REN for abortive treatment of migraine attacks in adolescents demonstrated persistent efficacy and showed a reduction in acute medications (16). Based on the recent randomized, double-blind, placebo-controlled trial of REN prevention in adults (10), the indication of REN was expanded for dual purposes and now includes both acute and preventive treatment.

However, the potential preventive benefits in adolescents have not been specifically reported previously. As such, the purpose of this real-world study is to assess the change in treatment days per month following the frequent acute use of the REN wearable device for the management of migraine in adolescents and to evaluate preventive benefits in this age group. The secondary outcomes include evaluating acute treatment efficacy in adolescent patients who get frequent treatment, as well as the assessment of the safety associated with these treatment patterns.

## Materials and methods

### Design and setting

The study (clinicaltrials.gov, NCT05769322) was a prospective, real-world evidence (RWE) analysis investigating adolescent users who have frequently used the REN wearable device at least 10 times for 1 month, tracking them over a 3-month period (see below). The primary outcome measure was the reduction in the mean monthly migraine treatment days (MMTD) for 3 months—from the first month of treatment and during two subsequent months. As a real-world evidence study assessing treatment efficacy in a clinical setting rather than in a research setting, a placebo arm was not included in the study, similar to a previous clinical trial involving REN in adolescents (15). Moreover, a meta-analysis comparing head-to-head and placebo-controlled trials found no significant long-term effects for migraine prophylaxis relative to placebo in pediatric patients (17).

### The Nerivio® REN device

**Figure 1:**
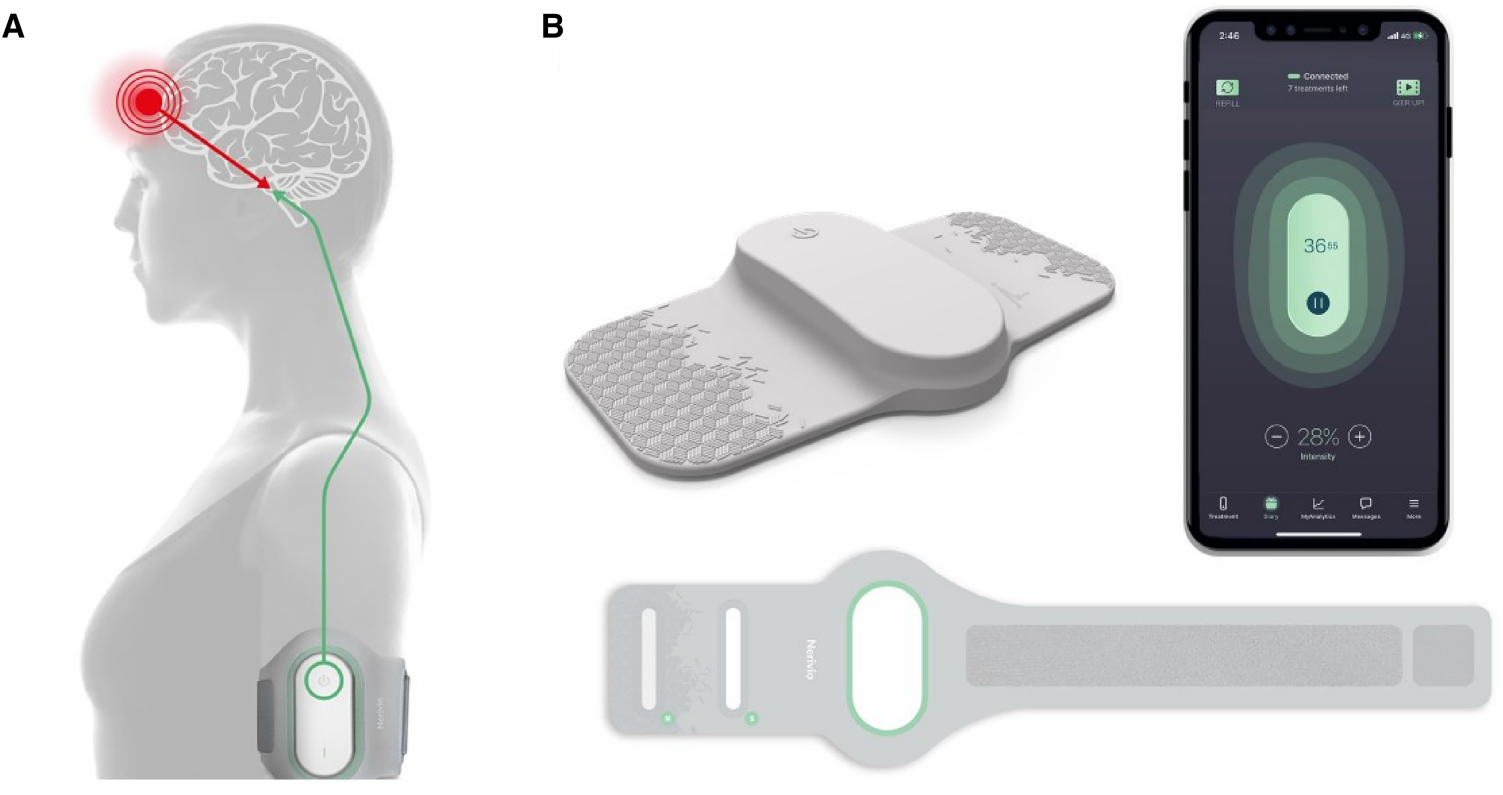
The REN wearable device (Nerivio®) and mechanism of action. (**A**) The REN wearable device provides neurostimulation for the acute and/or preventive treatment of migraine. Worn on the arm, it stimulates nociceptive Aδ and C fibers, triggering the CPM mechanism by sending information to the pain control center in the brainstem. This stimulation causes the release of endogenous neurotransmitters that cause a global pain inhibition response, suppressing the migraine headache pain and other associated symptoms. The thin device is secured to the arm via an armband and can be worn discreetly under a shirt. (**B**) The Nerivio is composed of the Nerivio device (top left), the armband (bottom), and the smartphone app (right).

The REN wearable device (Nerivio®) has been described in previous articles (8–16, 18). The device applies an electrical waveform designed to activate nociceptive receptors and thus to activate an endogenous pain mechanism, called conditioned pain modulation [CPM; previously known as diffuse noxious inhibitory control, DNIC; see (9, 10, 19, 20)]. The waveform is a symmetrical, biphasic, square pulse, with a modulated frequency range of 100–120 Hz, and an adjustable output current of up to 40 mA to the arm. This stimulation generates sub-painful nociceptive messages that activate the pain center in the brainstem, causing a global pain inhibition effect (Figure 1A). The device is a lightweight and thin non-invasive wearable that can be comfortably worn by all arm sizes, as it is applied to the upper arm with an adjustable arm band. The device itself features a single button at its center, which allows for a quick switch on. Once switched on, all treatment controls and interactions with the device are done through an easy-to-use smartphone application (app), which is connected to the device via Bluetooth (Figure 1B).

At the beginning of the treatment, the patients are instructed via the app to set the treatment intensity (output current) to a personal level that is strong but not painful, by increasing/decreasing the intensity with +/− buttons in the app. It takes a minute to wear the device, followed by 45 min of treatment, during which the patients may continue with their normal daily activity (study, play sports, participate in social activities, sleep, etc.), and are only refrained from immersing the device in large amounts of water (e.g., shower, pool). The compact design of the wearable device, which can be covered by a shirt, along with the smartphone control, makes it easy to use discreetly. Similar to a pack of pills with a set number of pills, each device unit is equipped with a battery that enables 18 treatment sessions. During treatments, the app displays the remaining treatment time, as well as the number of remaining treatments available within the device. The patients can order a refill device by pressing a refill button in the app.

### Data collection

During the registration process to the REN device, patients agree to the terms of use that state that personal information is voluntarily provided and that de-identified data may be used for research purposes. Information pertaining to the timing of treatments, treatment duration, and treatment intensity are automatically registered to a secure data server. Patients are prompted to answer questions to prospectively record their migraine attacks, accompanying symptoms, and the use of additional treatments. These data are then presented in a graphical summary and on a monthly calendar in the app, enabling patients to track their migraine and treatment patterns.

### Dataset

Real-world data of REN treatments were collected from adolescent patients, across the United States, who treated their migraine attacks with the REN wearable device between 1 January 2021 and 15 January 2023. The inclusion criteria were as follows: (1) adolescents, aged 12–17 years, as determined by the date of birth entered during the app registration; (2) frequent users defined as users who used the REN device on at least ten treatment days in 1 month of 28 days; (3) at least three treatments in each of the two consecutive months (months 2 and 3; 28 days each). A treatment day was defined as a day with a minimum treatment duration of 30 min. The usage pattern was chosen to resemble that of the REN preventive usage modality. The criteria of at least 10 treatment days in the first month followed by at least three treatment days in the following month (i.e., at least 13 treatments) assured that patients used at least two REN devices over these months, as each REN device provided 12 treatments at the time of data collection. By applying these criteria, the potential bias of reduction in the number of treatments due to a desire to save treatments and thus avoid refilling the prescription for another device, or the lack of an available device, is eliminated.

### Outcome measures

#### Primary endpoint: monthly migraine treatment days

The primary endpoint, MMTD, was calculated for each month, defined as a day on which a patient treated their migraine with REN. Treatment days per month was used as a proxy measure for the number of monthly migraine days (MMD), and the change between the mean number of MMTD from the first month (taken as “baseline”) to each of the following months was used to evaluate the presence and size of potential preventative benefits. A paired *t*-test was used to test for significant differences between MMTD.

#### Secondary endpoints

##### Efficacy

The secondary endpoints included treatment efficacy. Pain intensity was rated on a four-point scale: severe, moderate, mild, or none, and was voluntarily reported at the treatment baseline and 2 h after the treatment. Similarly, functional disability was rated on a four-point scale: severe limitation, moderate limitation, some limitation, or no limitation. Associated symptoms (photophobia, phonophobia, and nausea/vomiting) were marked as present or not. Evaluable treatments for efficacy analysis were those in which pain or functional disability were reported at both baseline and 2-h post-treatment, and those in which associated symptoms were reported as present at baseline and their presence/absence were reported at 2-h post-treatment. In all cases, an additional criterion was the absence of any reported usage of medications. The efficacy across multiple treatments was determined based on the proportion of adolescent frequent users who achieved a response to treatment from baseline to 2-h post-treatment (for each specific measure) in at least 50% of their treatments. The following secondary efficacy outcome measures are used: (1) consistent pain relief—decrease in headache pain from severe or moderate at baseline to mild or no pain at 2 h post-treatment; (2) consistent pain freedom—decrease in headache pain from severe, moderate, or mild at baseline to no pain at 2 h post-treatment; (3) consistent improvement in function—improvement of at least one grade between baseline and 2 h post-treatment, for treatments in which limitation was reported at baseline; (4) consistent return to normal function—a report of no functional disability at 2 h, for treatments in which limitation was reported at baseline; (5) freedom from photophobia, for treatments in which photophobia was reported at baseline; (6) freedom from phonophobia, for treatments in which phonophobia was reported at baseline; (7) freedom from nausea/vomiting, for treatments in which nausea/vomiting was reported at baseline; and (8) freedom from at least one associated symptom, for treatments in which one or more associated symptom(s) were reported at baseline.

##### Safety

All adverse events (AEs) that were reported within the study's period were analyzed, and the following information is provided: number of device-related AEs, their description and severity, and whether they were serious.

## Results

### Patients’ cohort

A total of 83 high-frequency adolescent users were found eligible for this analysis, reflecting the previously mentioned inclusion criteria and definition of frequent users. The users were 15.9 ± 1.3 years of age (mean ± SD), and 89% of them were female. A total of 2,834 treatments were conducted using the REN wearable device during the 3-month period under evaluation. A total of 81 adolescent frequent users reported the presence or absence of aura and associated migraine symptoms through a post-treatment questionnaire in the Nerivio® app administered 2 h after each treatment, in 1,458 of their treatments. The vast majority of the participants (77 out of 81 individuals, 95.1%) reported experiencing aura and/or one migraine-associated symptom on at least one occasion. Table 1 shows a breakdown of the aura and migraine-associated symptoms reported. Photophobia and phonophobia were most common, and over one-third of the users reported having aura.

**Table 1:** Presence of aura and associated migraine symptoms.

	Aura	Nausea	Photophobia	Phonophobia	At least one migraine symptom/aura
Users (% out of *n* = 81)	37 (45.7%)	61 (75.3%)	74 (91.4%)	71 (87.7%)	77 (95.1%)

Number (and percent) of users who reported the presence of aura and/or migraine-associated symptoms at least once.

### Primary endpoint: monthly migraine treatment days

**Figure 2:**
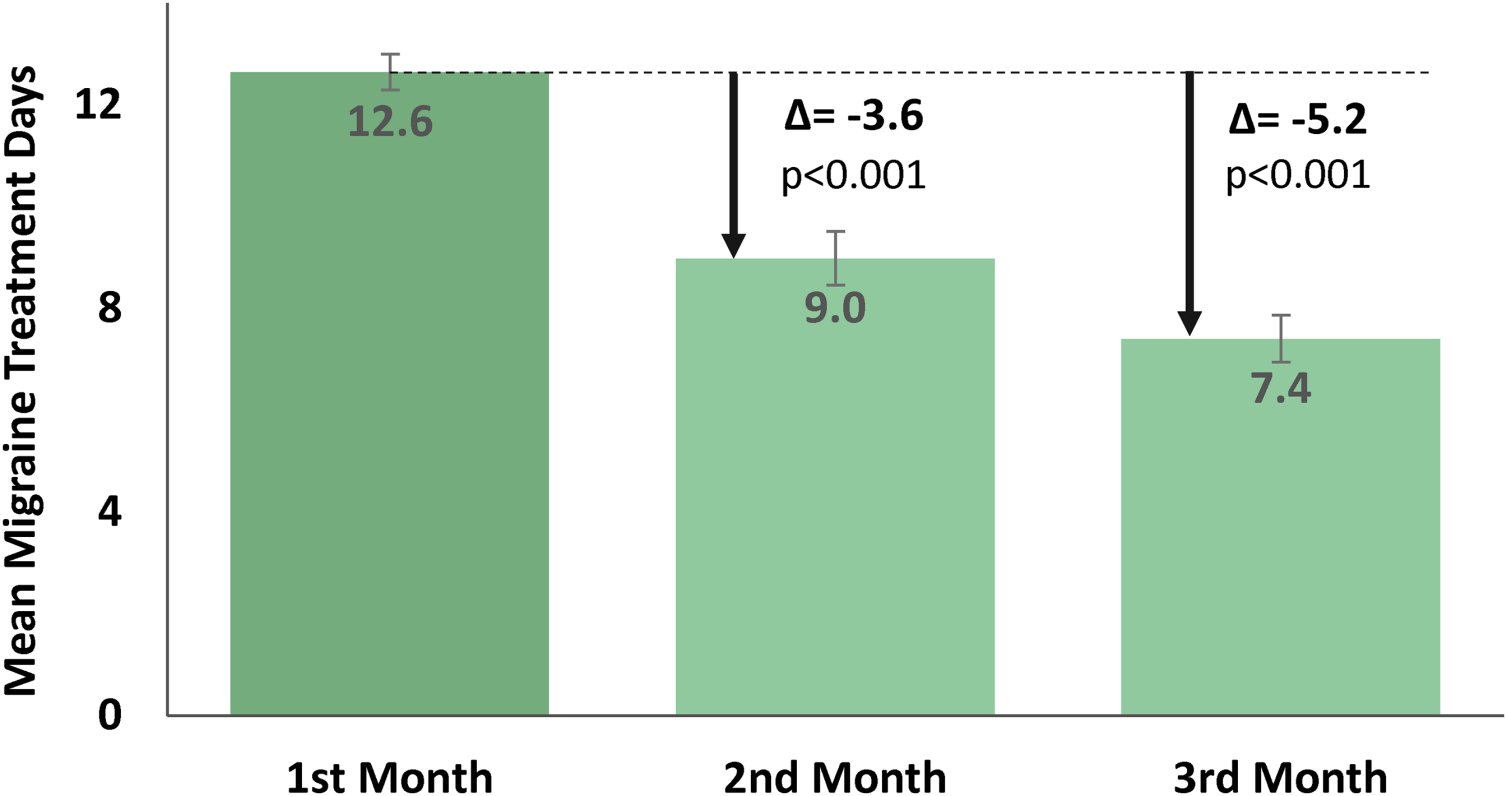
Reduction in mean MMTD, shown as mean ± SE.

The results (Figure 2) showed a significant month-to-month decrease in the primary endpoint of mean [± SD, (min–max)] MMTD. From 12.6 [±3.2, (10–23)] REN MMTD in the first month, MMTD dropped to 9.0 [±4.8, (3–25)] in the second month of consecutive use [*t*(82) = 7.0, *p* < 0.001, paired *t*-test], reflecting a reduction of 3.6 (±4.8) MMTD in the second month of treatment. The number of MMTD further decreased to 7.4 [±4.2, (3–19)] MMTD in the third month [*t*(82) = 3.5, *p* < 0.001, paired *t*-test], reflecting an additional reduction of 1.6 (±4.1) MMTD in the third month of treatment. A cumulative decrease of 5.2 (±4.8) MMTD was observed throughout the course of 3 months of REN treatment.

### Secondary endpoints: efficacy

**Figure 3:**
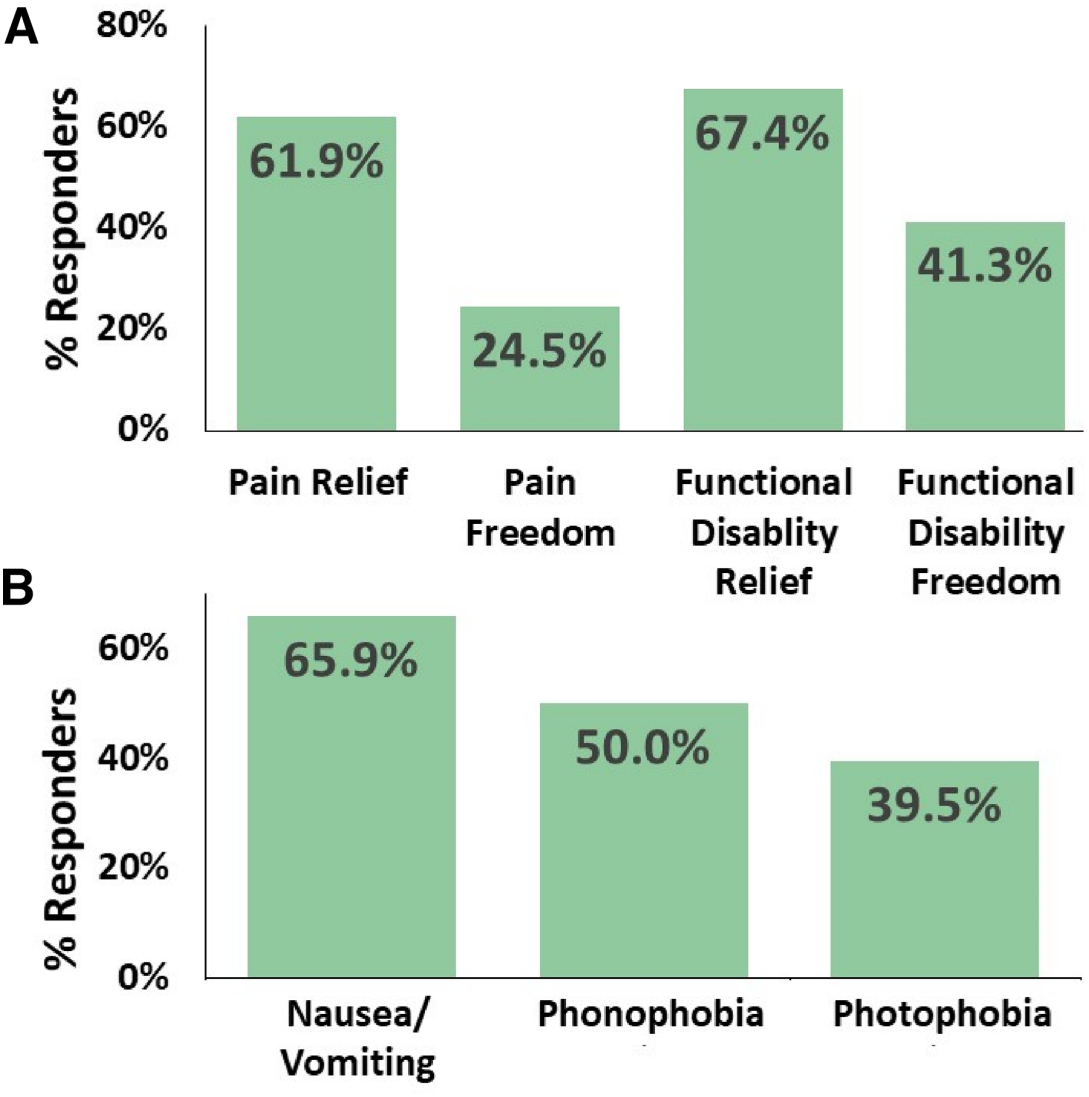
Consistent efficacy. The percentage of adolescents with migraine who achieved consistent efficacy at 2-h post-treatment in at least 50% of their treatments. (**A**) Relief of headache pain, freedom from headache pain, relief of functional disability, and freedom from functional disability. (**B**) Disappearance of associated symptoms when presented at baseline.

The secondary efficacy endpoints (Figure 3) showed a consistent acute pain response over a 2 h period in at least 50% of the treated attacks, with 61.9% (26/42) of adolescent frequent users reported pain relief, 24.5% (12/49) achieved pain freedom, 67.4% (31/46) experienced relief from functional disability, and 41.3% (19/46) achieved functional disability freedom. Moreover, REN resulted in a consistent 2-h disappearance of associated symptoms in at least 50% of the attacks in which a symptom was reported at the beginning of the treatment, with disappearance of photophobia in 39.5% (15/38) of the cases, the disappearance of phonophobia in 50.0% (17/34) of the patients, the disappearance of nausea/vomiting in 65.9% (14/23) of the cases, and the disappearance of at least one associated symptom in 65.9% (27/41) of the adolescent patients.

### Secondary endpoints: safety

In terms of safety, there was a single report of a minor, device-related AE, in which the user reported arm pain that subsequently resolved after the treatment. Following this event, the user continued to use the device and refilled their prescription for additional devices. There were no systemic or serious AEs.

## Discussion

Real-world evidence shows that among adolescents who treat their migraine attacks frequently with 10 or more REN treatment days per month, there is a significant reduction in the number of treatment days in each of the two following months. Moreover, the REN treatments are efficacious, leading to the freedom or relief from headache pain, associated symptoms, and functional disability, while being extremely safe. The treatment regimen observed in this study of an average of 12.6 treatment days in the first month of REN treatment (and more than 10 treatment days per patient in the first month) resembles the treatment protocol used in a recent pivotal, randomized, placebo-controlled trial that assessed the efficacy of REN for migraine prevention in adults (10). The results of the adults study indicate that when analyzing the modified intention to treat data (mITT; REN group: *n* = 95, placebo group: *n* = 84), it was observed that individuals who underwent REN treatments for 2 months, following an every-other-day treatment protocol, and maintained at least 86% adherence rate to the protocol (i.e., at least 12 treatment days per 28-day month), experienced a significant decrease of 4.0 (±4.0) mean (±SD) MMD in the REN group, whereas the placebo group only experienced a decrease of 1.3 (±4.0) MMD. A therapeutic gain of 2.7 MMD (*p* < 0.001) was achieved by the REN group. In the current study, the change in the mean number of MMTD from the first month, which is taken as “baseline,” to each of the following months was used to evaluate the presence and size of potential preventive benefits. The frequent use of REN for the acute treatment of migraine in a pattern and frequency similar to every-other-day, was shown to reduce the mean number of MMTD used by adolescents in two subsequent months, by 3.6 and 1.6 MMTD, respectively, suggesting potential preventive benefits. Even when not fully adhering to the every-other-day treatment protocol, adults with less than 12 treatment days per 28-day month in the intention to treat (ITT) group of the pivotal study (10) achieved preventive benefits from REN, suggesting that 9.0 MMTD in the second month of treatment in the current study could contribute to the additional preventive benefit seen in the third month of the study.

The preventive management of migraine in adolescence is largely extrapolated from adult studies, given the limited availability of randomized controlled trials and approved or cleared options by the FDA in this patient population. There are no medications approved for migraine prevention in adolescents that have obtained level A evidence (2). The AAN practice guidelines for pediatric migraine prevention gives a level B recommendation to only three treatments: topiramate, propranolol, and amitriptyline combined with cognitive behavior therapy (2). Of these, only topiramate is FDA-approved in adolescents 12 years of age and above, meaning that a large part of pediatric and adolescent migraine prophylaxis is off-label (2, 21). According to the latest consensus guideline on preventive treatment for adolescents (2), the combination of amitriptyline (a tricyclic antidepressant) plus cognitive behavioral therapy (CBT) was the only migraine prevention treatment option for adolescents supported by high-confidence evidence of efficacy.

Pharmacological treatment of migraine in adolescents is challenging for reasons beyond a paucity of evidence-based and FDA-approved options. Many teenagers and their parents are reluctant to use medication at all and prefer a “more natural” approach. Some individuals may discontinue treatment due to the adverse effects of the medication being perceived as more severe than the migraine itself (16). Treatment non-adherence is also a major concern in pediatrics in general, with approximately 65%–90% of adolescents being non-adherent to treatment protocols (22). More specific to migraine, studies have shown that the medication adherence rates of daily preventive medication in adolescents are 64%, as reported by the individuals themselves on a daily basis (22). Adherence to migraine prevention medications in adults is as low as 26.6%−32.4% after 6 months of migraine prevention medications, according to a large database of health insurance claims in the United States. This finding indicates that many patients discontinue the use of their prescribed medications at 6 months (23). Importantly, the adherence to migraine prevention treatment can be increased with the use of electronic monitoring (22, 24).

It is recommended that all patients with migraine be provided with acute treatment plans to alleviate the symptoms of an attack. However, abortive medication for migraine in adolescents may carry significant challenges. Many schools enforce regulations that prohibit students from possessing their own medication, instead mandating that such medications be dispensed by a nurse in adherence to a physician-prescribed plan that is kept on file. Some students are unable or unwilling to leave class to visit the school nurse and therefore wait until school ends, leading to delays in treatment and potentially more treatment-resistant pain (25). The availability of FDA-approved and efficacious acute medications for migraine in adolescents is currently restricted, with only four types of migraine-specific drugs that have been approved for abortive treatment of migraine in patients aged 12–17 years (rizatriptan, almotriptan, sumatriptan–naproxen, zolmitriptan) (16). However, some adolescents become frustrated by suboptimal medication efficacy, bothersome side effects, or the burden of frequent pill-taking, and prefer to work through the pain. On the other hand, those who overuse abortive medication to manage frequent migraine attacks, whether OTC medications or migraine-specific triptans, are at risk for developing medication overuse headache (MOH), which can lead to higher frequency and intensity of migraine attacks (26). In contrast to frequent use of pharmaceutical abortive agents, frequent use of REN is not associated with overuse headache syndromes. Moreover, the substantial therapeutic efficacy including the consistency and persistent effects of frequent use of REN for acute migraine attacks act as neuromodulatory effects, leading to relief and freedom from headache pain and of functional disability, which are critical for adolescents’ quality of life. Furthermore, the use of REN leads to the improvement of migraine-associated symptoms. The most common associated symptom is photophobia, while the least common associated symptom is nausea/vomiting, which benefits the most from REN. Taken together, this device may prevent the progression of migraine associated with inadequate treatment, a recognized risk factor for chronic migraine (27).

As such, there is a significant demand for non-pharmaceutical FDA-cleared options for abortive and preventive management of migraine for adolescents that are efficacious, tolerable, and safe. Vagus nerve stimulation (nVNS) is a prescribed, non-wearable device that is placed on the neck to stimulate the vagus nerve to block pain signals. The device is FDA-cleared for the preventive treatment of migraine headache in adolescent and adult patients, and for the acute treatment of pain associated with migraine headaches in adolescent and adult patients (28). Single pulse transcranial magnetic stimulation (sTMS) is a non-wearable device placed on the back of the head to block cortical spreading depression (29, 30), FDA-cleared for the acute and prophylactic treatment of migraine headaches in adolescents and adults. In contrast, REN is a wearable device, which patients do not need to hold in order to use. It has been shown to be highly effective, well-tolerated, and safe in a clinical trial evaluating the acute treatment of migraine in adolescents (15). A *post-hoc* analysis comparing REN with standard care medications demonstrated that REN might be more efficacious for adolescents than some acute pharmacological medications (18). REN was previously FDA-cleared for the *acute* treatment of migraine in adults and adolescents. Based on a placebo-controlled pivotal study of migraine prevention in adults (10) and the data presented in this article, REN is now *also* cleared for migraine *prevention* in adults and adolescents, as of 6 February 2023 (31). While wearable sensor technology for pain detection is not currently available for any of the non-invasive FDA-cleared neurostimulation devices, the Nerivio® app allows for tracking of migraine attacks and their intensity through electronic diaries.

The REN wearable device has multiple strengths. It is associated with high efficacy, good tolerability, and a favorable safety profile, with no systemic or serious side effects. As aforesaid, the device can be used discreetly, giving adolescents the freedom of treating their migraine attacks during school classes, without needing to leave the classroom and go to the school nurse, empowering adolescents with the feeling of control and autonomy. Being connected to a smartphone app allows adolescents intuitive, easy, and accessible control of their migraine disease. Almost all teens (aged 13–17) in the United States use a smartphone daily [95% as of 2022 according to Pew Research Center (32)], and use it for many hours per day. The app moreover provides unique features that are appealing and adopted by adolescents. For instance, in-app reminders help adolescents adhere to the migraine prevention treatment (24), with the ability to personalize the reminders to the hour of the day and to various days (e.g., every even or odd day of the month, or on even or odd weekdays and one weekend day).

Migraine guidelines and recent consensus statements suggest that behavioral interventions may be helpful for migraine management and may be helpful for common migraine comorbidities such as anxiety, depression, and insomnia (2, 33, 34). Furthermore, the app has a specially tailored behavioral therapy consisting of Guided Intervention of Education and Relaxation (GIER). GIER includes relaxation of diaphragmatic breathing, progressive muscle relaxation, guided imagery, and education on migraine biology and REN mechanism. Combining the GIER behavioral intervention with REN treatment was shown to improve the therapeutic efficacy beyond that of REN alone (35), providing an additional feature that can benefit adolescents. Finally, as a device that is worn on the arm, the users have less risk of intolerance due to allodynia, which is especially common in the head—the main body part affected by allodynia during migraine attacks (36).

Another key benefit of REN prevention comes from its associated cost-saving aspects. REN prevention leads to a significant reduction in the utilization of acute medications and healthcare provider appointments among adults, resulting in a mean annual cost-saving of $5,854 per patient (37). REN prevention further leads to an additional annual cost-saving of $4,146 from work-related activities (absenteeism and presenteeism days) in adults. While work-related cost-savings is irrelevant for adolescents, a major hurdle of adolescents with migraine comes from missed school and social activities (7), which are also expected to be reduced with REN prevention.

The study has a few main limitations. First, the number of MMD was not measured directly but derived from the number of abortive MMTD, and preventive effects were extrapolated from using the device for acute treatment and not directly for migraine prevention. However, given that the exact same stimulation is used for both abortive and preventive treatment and that the frequency of treatments met the usage pattern in the prevention pivotal trial in adults, deriving MMD from MMTD is clinically meaningful. Second, as a post-marketing surveillance study, the cohort was selected from the users treated with the REN device, presumably reflecting that those who found it useful were likely to use it more. To directly assess preventive benefits from treating with the REN wearable device in adolescents, further research is needed with a pre-planned clinical trial including those who require migraine prevention treatment and will report their migraine attacks in a daily migraine diary (migraine days), which is available in the Nerivio® app. A dedicated study will further allow the collection of patient-centered outcomes, such as treatment satisfaction and quality of life. Third, frequency swings in the number of monthly migraine attacks are quite common, particularly in patients with chronic migraine (38), and thus using a single month for migraine baseline assessment may be short. However, 1 month is the most common baseline period used in migraine studies, including previous REN studies. Moreover, the reduction in number of MMTD between the first and third months was larger than the overall standard deviation of MMTD over all users during the three study months, indicating a larger effect of MMTD reduction over that of frequency swings, thus suggesting that the reduction of MMTD due to an efficacious REN treatment overcomes the natural fluctuations in migraine frequency. An extended study, tracking adolescents for more treatment months, will shed more light on the long-term efficacy of REN for migraine prevention in adolescents. Lastly, the patients in the present study had a high attack frequency, which is a known risk factor for migraine chronification (39), and is associated with the sensitization of migraine-related structures (40). As abovementioned, the wearable REN device activates an endogenous pain mechanism, the CPM, to abort attacks and preventive migraine days. However, there is a need for investigations designed to elucidate the underlying central mechanisms that drive the observed therapeutic clinical effects of migraine prevention with REN, and specifically the potential of brain reorganization and neuroplasticity.

## Conclusion

The development of safe and effective wearable technologies with the capacity for monitoring outcomes represents a major therapeutic advancement in pain management. The frequent use of the REN wearable device for the acute treatment of migraine was shown to reduce the mean number of monthly treatments in adolescents, with a similar reduction in migraine and headache days seen in a pivotal prevention trial in adults. This real-world REN data supports the broader application of the dual acute and preventive indication, given the safety profile, potential preventive benefits, as well as the positive acute efficacy data for the treatment of migraine in adolescents. Further studies are needed to assess the long-term impact on migraine-related disability and quality of life of adolescents using REN for migraine prevention.

## Data Availability

The datasets generated during and/or analyzed during the current study are available from the corresponding author on reasonable request.
